# *Ex vivo* gut cultures of *Aedes aegypti* are efficiently infected by mosquito-borne alpha- and flaviviruses

**DOI:** 10.1128/spectrum.05195-22

**Published:** 2023-08-04

**Authors:** Ana Lucia Rosales Rosas, Lanjiao Wang, Sara Goossens, Arno Cuvry, Li-Hsin Li, Nanci Santos-Ferreira, Alina Soto, Kai Dallmeier, Joana Rocha-Pereira, Leen Delang

**Affiliations:** 1 KU Leuven Department of Microbiology, Immunology and Transplantation, Rega Institute for Medical Research, Laboratory of Virology and Chemotherapy, Leuven, Belgium; 2 KU Leuven Department of Microbiology, Immunology and Transplantation, Rega Institute for Medical Research, Molecular Vaccinology and Vaccine Discovery, Leuven, Belgium; Centro de Investigacion y de Estudios Avanzados del Instituto Politecnico Nacional, Mexico City, Mexico

**Keywords:** arbovirus, mosquito gut, *ex vivo*, antivirals

## Abstract

*Aedes aegypti* mosquitoes can transmit several arboviruses, including chikungunya virus (CHIKV), dengue virus (DENV), and Zika virus (ZIKV). When blood-feeding on a virus-infected human, the mosquito ingests the virus into the midgut (stomach), where it replicates and must overcome the midgut barrier to disseminate to other organs and ultimately be transmitted via the saliva. Current tools to study mosquito-borne viruses (MBVs) include 2D-cell culture systems and *in vivo* mosquito infection models, which offer great advantages, yet have some limitations. Here, we describe a long-term *ex vivo* culture of *Ae. aegypti* guts. Cultured guts were metabolically active for 7 d in a 96-well plate at 28°C and were permissive to ZIKV, DENV, Ross River virus, and CHIKV. *Ex vivo* guts from *Culex pipiens* mosquitoes were found to be permissive to Usutu virus. Immunofluorescence staining confirmed viral protein synthesis in CHIKV-infected guts of *Ae. aegypti*. Furthermore, fluorescence microscopy revealed replication and spread of a reporter DENV in specific regions of the midgut. In addition, two known antiviral molecules, β-d-N^4^-hydroxycytidine and 7-deaza-2′-C-methyladenosine, were able to inhibit CHIKV and ZIKV replication, respectively, in the *ex vivo* model. Together, our results show that *ex vivo* guts can be efficiently infected with mosquito-borne alpha- and flaviviruses and employed to evaluate antiviral drugs. Furthermore, the setup can be extended to other mosquito species. *Ex vivo* gut cultures could thus be a new model to study MBVs, offering the advantage of reduced biosafety measures compared to infecting living mosquitoes.

**IMPORTANCE:**

Mosquito-borne viruses (MBVs) are a significant global health threat since they can cause severe diseases in humans, such as hemorrhagic fever, encephalitis, and chronic arthritis. MBVs rely on the mosquito vector to infect new hosts and perpetuate virus transmission. No therapeutics are currently available. The study of arbovirus infection in the mosquito vector can greatly contribute to elucidating strategies for controlling arbovirus transmission. This work investigated the infection of guts from *Aedes aegypti* mosquitoes in an *ex vivo* platform. We found several MBVs capable of replicating in the gut tissue, including viruses of major health importance, such as dengue, chikungunya, and Zika viruses. In addition, antiviral compounds reduced arbovirus infection in the cultured gut tissue. Overall, the gut model emerges as a useful tool for diverse applications such as studying tissue-specific responses to virus infection and screening potential anti-arboviral molecules.

## INTRODUCTION

Arboviruses are a diverse group of viruses that rely on arthropod vectors such as mosquitoes, ticks, and sandflies to infect susceptible vertebrate hosts and perpetuate transmission. Three arboviral families/orders are of most clinical relevance: *Bunyavirales*, *Flaviviridae*, and *Togaviridae*, as they encompass important arboviruses of global health concern, such as La Crosse virus, dengue virus (DENV), Zika virus (ZIKV), and chikungunya virus (CHIKV) (1). These are mosquito-borne viruses (MBVs) and are mainly transmitted by female *Aedes* (*Ae*.) mosquitoes (e.g., *Ae. triseriatus, Ae. aegypti,* and *Ae. albopictus*) (2), with *Ae. aegypti* mosquitoes considered highly competent vectors because of their anthropophilic behavior (3).

When blood-feeding on a virus-infected human or animal, a female mosquito will ingest the virus-containing blood in the midgut (i.e., mosquito stomach), where the virus must replicate and overcome the midgut barrier to subsequently disseminate to mosquito secondary organs. As the mosquito becomes systemically infected, resulting in a high viral load, the virus will reach the salivary glands. The virus will be released in the saliva upon the next bite, allowing virus transmission when the mosquito feeds on uninfected hosts (4, 5). The midgut is thus the initial site for infection and replication of any arbovirus in the mosquito. Virus replication in and spread across midgut cells is a requisite for a productive arbovirus infection in the mosquito (4).

*In vitro* cell cultures can be useful to study arbovirus infection and other relevant processes in mosquitoes; however, they present several limitations. Most of the established mosquito-derived cell lines are not derived from tissues relevant to specific stages of mosquito-virus interactions (e.g., salivary glands or midgut) but were generated from larval or embryonic tissue [e.g., *C6/36* (larvae) and *Aag-2* (embryos) cells] (6). Therefore, results obtained from *in vitro* experiments ought to be taken cautiously. Furthermore, cell lines are considered homogeneous cultures, both genetically and phenotypically, and single-cell type populations might not well represent multicellular tissues such as a midgut (7, 8).

Another method commonly used to study mosquito-virus interactions is the mosquito infection model. Through this approach, mosquito infection occurs artificially via a bloodmeal (membrane filled with infectious blood), and mosquitoes are sacrificed to assess viral infection and vector competence (9). Although living-infected mosquitoes provide valuable transmission data, the value of these data is directly proportional to the labor-intensive nature of the method. Transmission studies require skilled personnel for handling of infected mosquitoes and facilities operating at a high biosafety containment level, and carry the risks of personnel prick injury with virus-infected dissection tools or of personnel infection by a mosquito bite when an infected mosquito would escape from their containment receptacle within the facility.

*Ex vivo* organ cultures have been arising as an advantageous tool in research and have also been described for insects. For instance, *ex vivo* culture of insect organs has been successfully and widely defined for ixodid ticks. The synganglion, midgut, and salivary glands dissected from ticks were viable for 10 d and permissive to both Langat and Powassan viruses (8, 10). Reports on *ex vivo*/*in vitro* organ cultures from mosquito tissues are limited, but mainly focused on germline, gut, fat body, and carcass tissues (11 – 13). Previously, mosquito gut cultures have been a useful tool to address physiological processes, such as defining the role of certain neuropeptides in the mosquito immune response (14). Commonly, the duration of these gut cultures ranged from 24 to 35 h in incubation, except for one study that maintained cultured mosquito guts for 5 d post-dissection (d.p.d.) to assess DNA synthesis (14 – 16). Despite the essential implication of the mosquito gut in arbovirus replication and dissemination, no *ex vivo* mosquito gut infection with an arbovirus has been adequately described.

Moreover, e*x vivo* cultures could offer a unique perspective to (i) study vector–virus interactions in a relevant tissue, for example, the mosquito gut, (ii) reveal tissue-specific responses to virus infection, or (iii) study tissue-specific metabolic processes. In addition, *ex vivo* mosquito gut cultures could provide a convenient and relevant platform to generate results that can be extrapolated to living mosquitoes. Finally, *ex vivo* gut cultures provide a controlled environment in which many parameters can be easily adjusted, and they constitute a safer option due to the low degree of containment required while working with BSL2/3 arboviruses, compared to working with live mosquito infection models.

On the lookout for novel tools that could be of use to study arbovirus infection within the arthropod vector, we dissected *Ae. aegypti* guts and established their viability for a 7-d period. We also infected the gut tissues with several mosquito-borne alpha- and flaviviruses, confirming the production of infectious viral particles. Furthermore, previously reported antiviral drugs were able to significantly reduce arbovirus replication in the treated guts. We thus developed an *ex vivo* mosquito gut model that constitutes a valuable tool to study MBVs which complements the arsenal available to the mosquito arbovirus research field.

## RESULTS

### Viability of *Ae. aegypti* guts cultured *ex vivo*

**Fig 1:**
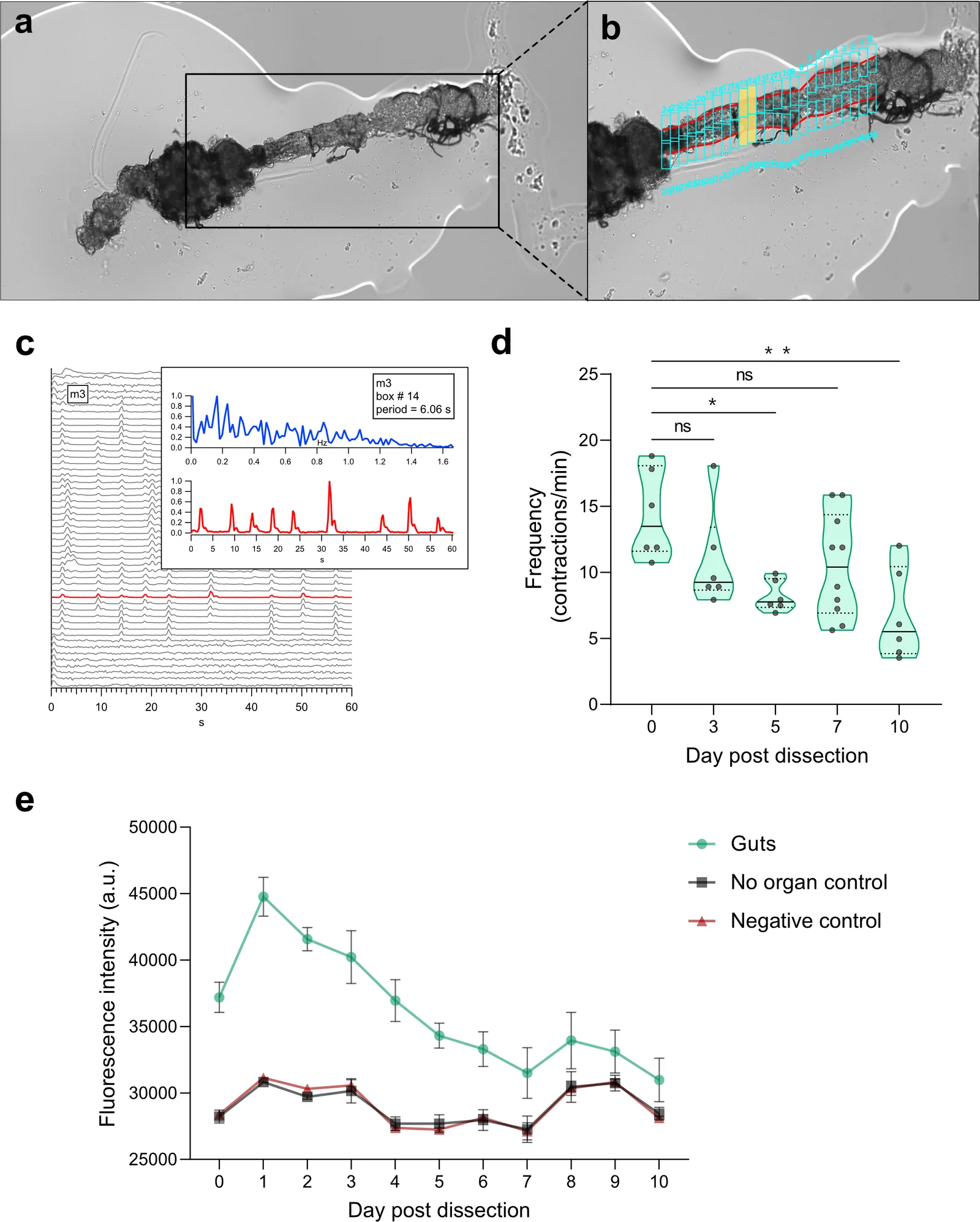
*Ex vivo* cultured mosquito guts were viable for 7 d.p.d., based on hindgut peristalsis and a resazurin-based assay. (**a**) Representative image of a mosquito gut placed in a carboxymethyl cellulose (CMC) drop for videography. (**b**) Close-up of the hindgut which comprises the area of analysis (AOA) manually set and outlined by the red lines. Cyan boxes denote the regions to be analyzed along the AOA. The number and size of the regions (cyan boxes) were adjusted to be 25 boxes on each side of the hindgut. (**c**) Representative kymograph showing the output for one mosquito gut analysis. Each line in the kymograph corresponded to an individual region, which was further investigated in a plot. In the figure, the red highlighted line in the kymograph correlates to the presented plot displaying a representative analysis readout for this region. (**d**) Contractions observed per minute as a function of time post-dissection in days. Each point represents one individual mosquito gut. The black line represents the median value. The graph shows data from three independent experiments. Statistical significance was assessed using the Kruskal–Wallis test. Significantly different values are indicated by asterisks: *, *P* = 0.0372; **, *P* = 0.0087. (**e**) Viability readouts exhibit metabolic activity for a period of 10 d for tested guts, no organ, and negative controls. Assays were performed with two or three biological replicates, each replicate composed of 3 guts per well, along with its corresponding controls. Error bars represent the standard errors of the means per time point. The graph shows data from three independent assays.

We first determined the viability of the dissected mosquito guts in 96-well culture plates. Since motility could be observed along the gut tissue following the dissection, a bioassay was performed based on the number of contractions by the hindgut (Supplementary material, Movie 1). The same area of the hindgut was analyzed for all biological replicates (Fig. 1b and c). Dissected guts presented peristalsis in the hindgut for up to 10 d.p.d. (Fig. 1d), indicating that the tissue was still viable after dissection. Notably, at 5 d.p.d., there was a significant decrease in the frequency of contractions per minute observed compared to *ex vivo* guts at 0 d.p.d. (mean values, day 0: 14.37 s vs day 5: 8.20 s). However, when measured at 7 d.p.d., the mean frequency was not significantly different from day 0 values (day 0: 14.37 s vs day 7: 10.49 s). Last, at 10 d.p.d., the mean frequency in the *ex vivo* cultured guts was strongly reduced compared to day 0 measurements (day 0: 14.37 s vs day 10: 6.74 s) (Fig. 1d), suggesting that the tissue’s viability had decreased over time.

We further assessed the metabolic activity of the *ex vivo* cultured gut tissue in a resazurin salt-based assay. The dissected guts remained metabolically active up until 7 d.p.d. (Fig. 1e), corresponding to the data obtained from hindgut peristalsis. Fluorescence intensity in the tested guts was still slightly higher than the “no organ” (NO) controls (used to measure background signal) and negative controls [NCs, paraformaldehyde (PFA) fixed guts] at 8, 9, and 10 d.p.d. However, there was more variability among biological replicates, and therefore, 7 d.p.d. was selected as the end time point for the following experiments. Of note, metabolic activity measured with the resazurin salt-based assay was further confirmed by performing assays on individual guts measuring ATP as an indicator of viability (Supplementary material, Fig. S1).

In addition, the effect of the neurotransmitter serotonin and the anticholinergic drug atropine on peristalsis was assessed to rule out that this visual feature might be a reflex. Guts (day 0 post-dissection) were exposed to serotonin, atropine, or gut medium (mock exposure), after which their hindgut peristalsis was recorded. The mean frequency displayed by the mock-exposed guts was 7.349 s (five biological replicates, median value: 7.48 s). Guts exposed for 1 h to serotonin displayed a slight increase in hindgut motility, with a mean peristaltic period of 12.76 s (six biological replicates, median value: 10.61 s), whereas atropine-exposed guts exhibit a somewhat reduced peristalsis, with a mean peristaltic period of 6.72 s (six biological replicates, median value: 7.58 s). Despite the observed trends, there were no statistically significant differences among the conditions evaluated (Supplementary material, Fig. S2).

### Infection of dissected *Ae. aegypti* guts with mosquito-borne alpha- and flaviviruses

**Fig 2:**
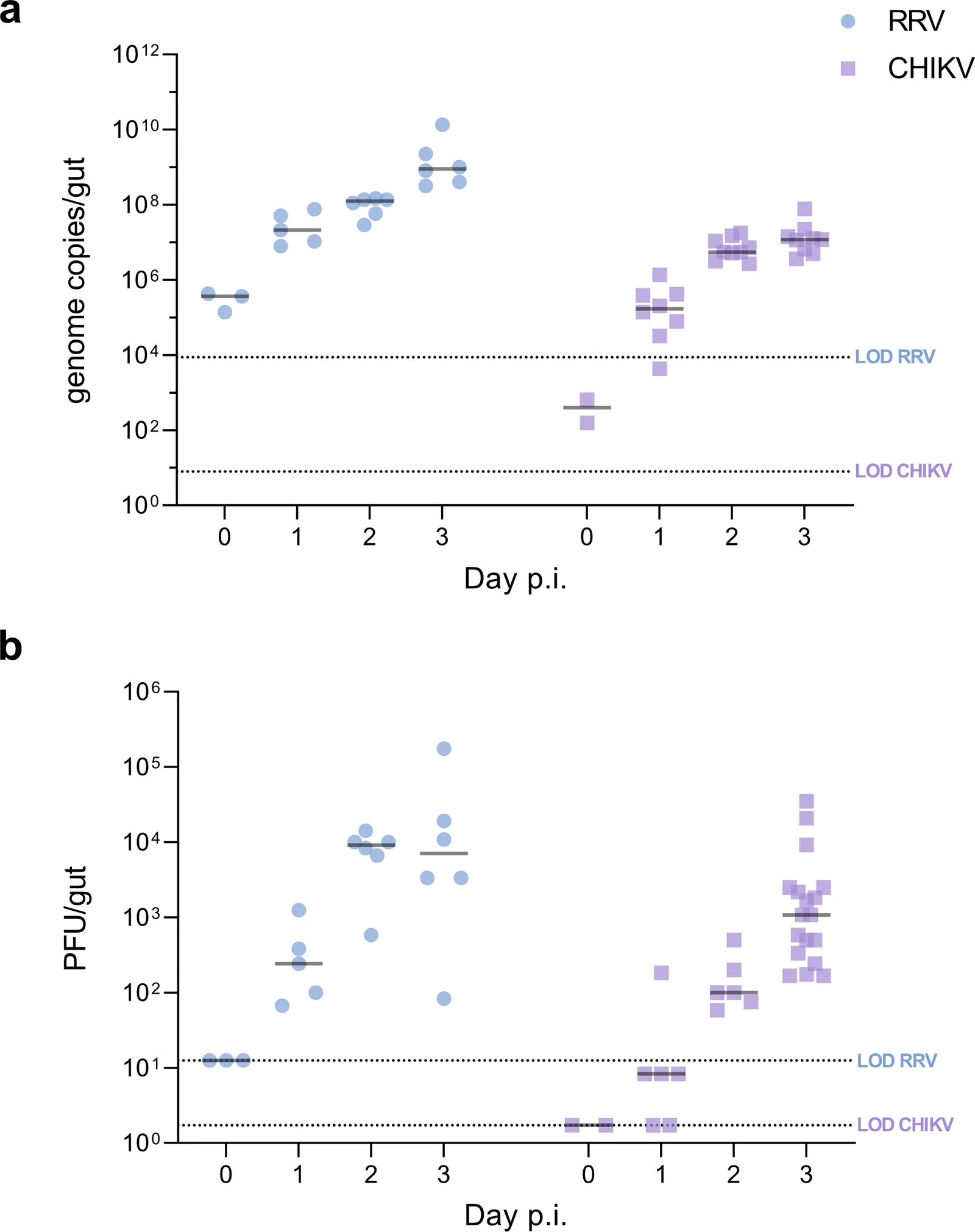
*Ex vivo* cultured guts supported RRV and CHIKV infection. (**a**) Viral RNA levels of RRV and CHIKV in the mosquito guts were quantified at 2 h (day 0), 1, 2, and 3 d.p.i. by means of qRT-PCR. (**b**) Infectious virus loads of RRV and CHIKV in the mosquito guts were quantified by means of plaque assay. Each symbol represents an individual gut organ. The black line represents the median value. Data correspond to at least two independent replication kinetics assays. LOD: Limit of detection of the corresponding assay.

Next, *ex vivo* virus infection was performed in the cultured guts to test their capacity to support the replication of several arboviruses. Replication kinetics were evaluated for Ross River virus (RRV) and CHIKV as representative members of the alphavirus genus of the *Togaviridae* family (Fig. 2). Both viruses were able to efficiently replicate in the *ex vivo* cultured guts with an increase in viral RNA levels between 1 and 3 d post-infection (d.p.i.), compared to viral RNA levels at 2 h p.i. (h.p.i.). Infectious virus titers of RRV and CHIKV increased accordingly in the gut tissue, while no infectious virus was observed yet at 2 h.p.i. The peak of viral RNA and viral titer levels was detected at 3 d.p.i. for both alphaviruses.

**Fig 3:**
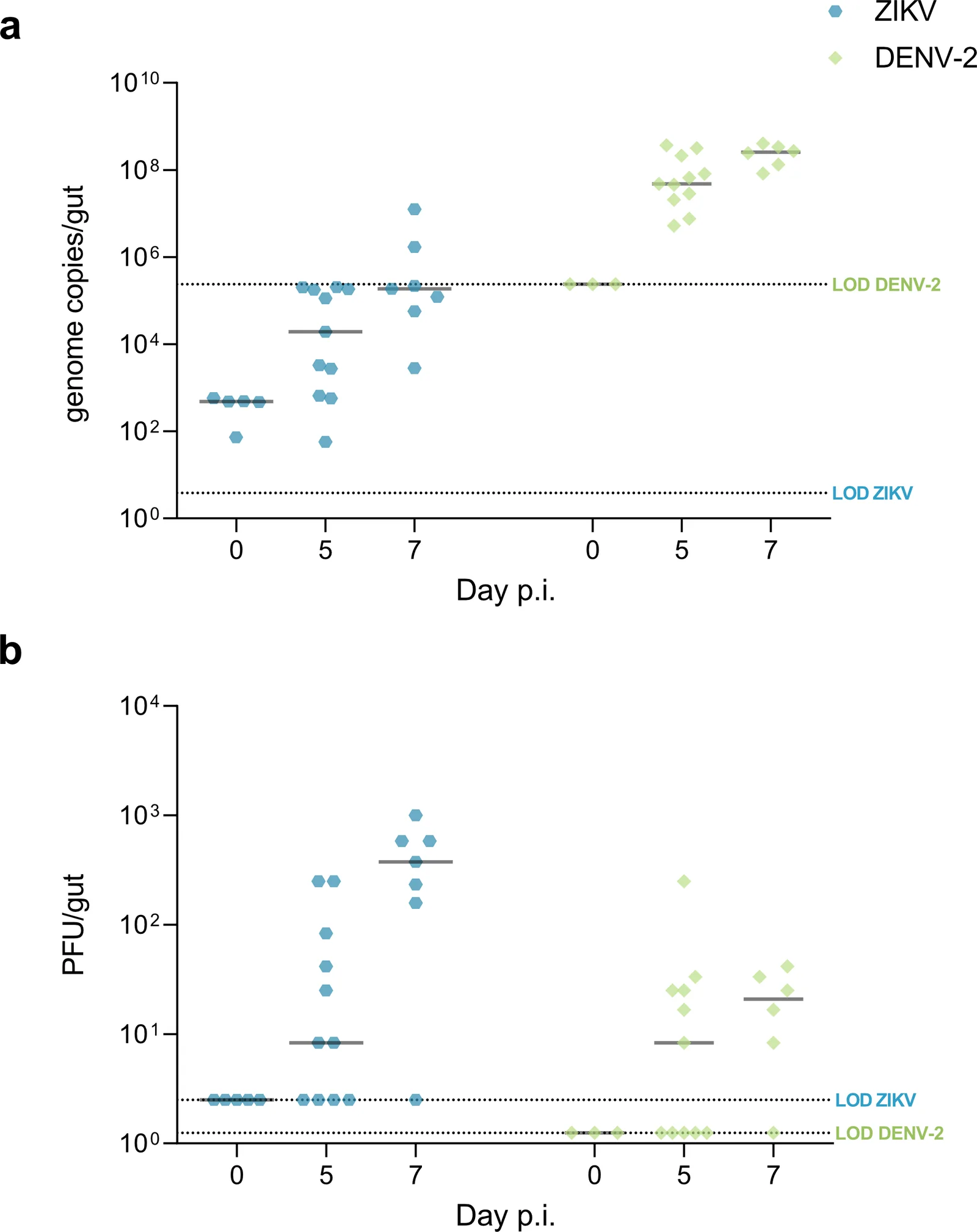
*Ex vivo* cultured guts supported DENV serotype 2 and ZIKV infection. (**a**) Viral RNA levels of DENV-2 and ZIKV in the mosquito guts were quantified at 2 h.p.i. and 5 and 7 d.p.i. by means of qRT-PCR. (**b**) Infectious virus loads of DENV-2 and ZIKV in the mosquito guts were quantified by means of plaque assay. Each symbol represents an individual gut organ. The black line represents the median value. Data correspond to two independent replication kinetics assays. LOD: Limit of detection of the corresponding assay.

To evaluate whether *Aedes*-borne flaviviruses could replicate in the *ex vivo* guts, the replication kinetics of DENV-2 and ZIKV were studied. As both viruses needed a longer period to replicate in the gut tissue (data not shown), viral RNA and infectious virus levels were measured starting at 5 d.p.i. (Fig. 3). Viral titers were increased at 5 d.p.i. and further peaked at 7 d.p.i., with DENV-2 reaching RNA levels up to 2.5 × 10^8^ genome copies/gut and a viral titer of 21 plaque forming unit (PFU)/gut (Fig. 3a). On the contrary, ZIKV replication and infection presented more variability among individual guts. The maximum amount of ZIKV RNA and infectious virus quantified at 7 d.p.i. were 2.1 × 10^6^ genome copies/gut and 4.2 × 10^2^ PFU/gut, respectively (Fig. 3b).

### Usutu virus can modestly replicate in *ex vivo* cultured guts from *Culex pipiens* mosquitoes

**Fig 4:**
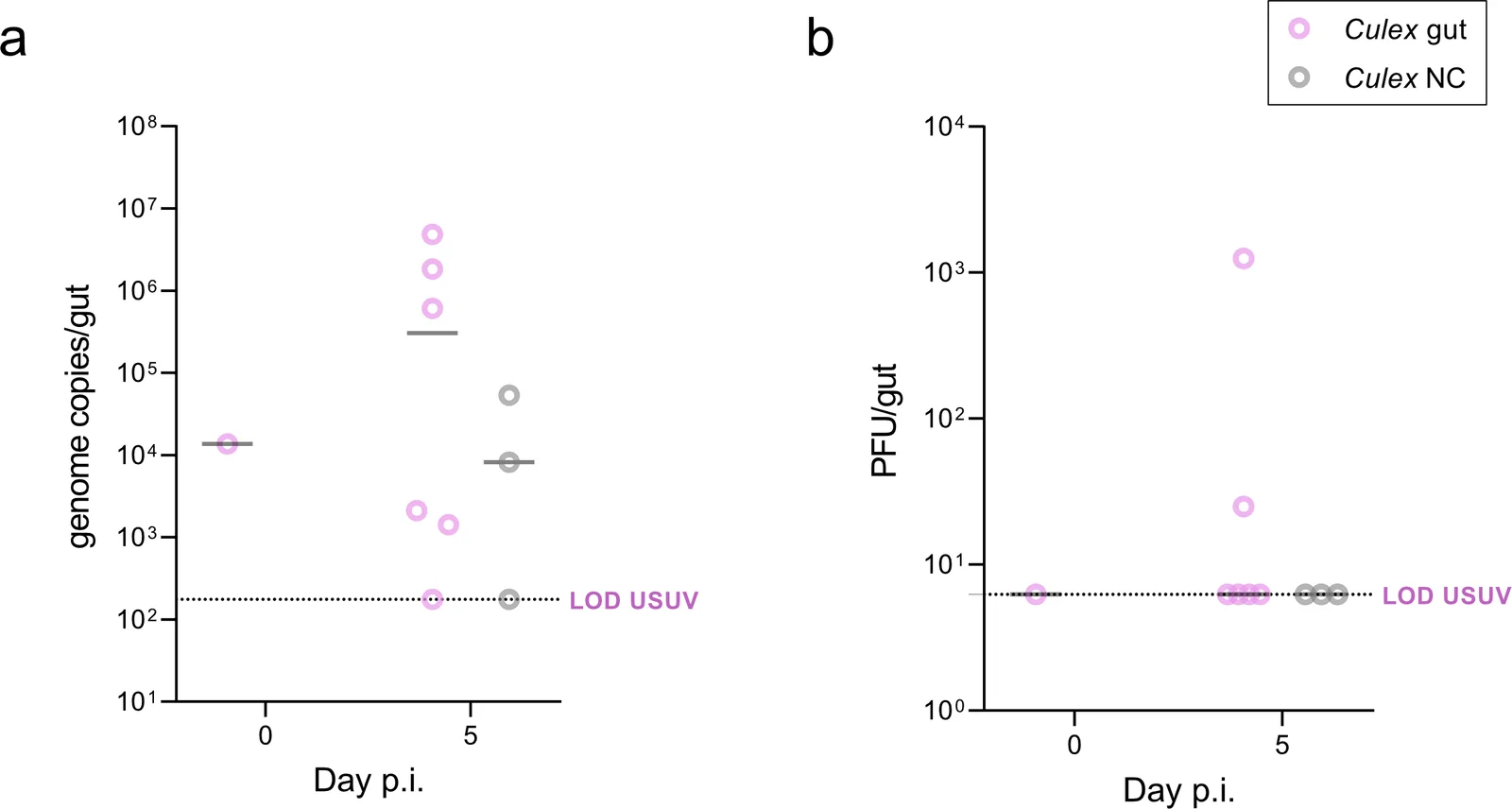
*Ex vivo* cultured guts of *Cx. pipiens* were partially permissive to USUV infection. (**a**) Viral RNA levels in the mosquito guts were quantified at 2 h (0 d.p.i.) and 5 d.p.i. by means of qRT-PCR. (**b**) Infectious virus loads in the mosquito guts were quantified by means of plaque assay. Each symbol represents an individual gut organ. The black line represents the median value. LOD: Limit of detection of the corresponding assay. NC, negative control.

As a proof-of-concept, the *ex vivo* gut model was applied to another mosquito species: *Culex (Cx.) pipiens*. The guts of *Cx. pipiens* thrived in culture and showed peristaltic movements during their incubation, as *Ae. aegypti* hindguts did. Therefore, we evaluated the susceptibility of these *Cx*. guts to Usutu virus (USUV), as *Cx. pipiens* mosquitoes are considered a competent vector for this virus (17). USUV infection was assessed in the guts at 7 d.p.i. by qRT-PCR and plaque assay. Only 50% (3 out of 6) of the guts became infected with USUV, reaching up to 1.8 × 10^6^ genome copies/gut. However, only two out of these three USUV-infected guts contained infectious virus, amounting to 6.4 × 10^2^ PFU/mL. Of note, a considerable amount of viral RNA was detected in samples corresponding to 2 h.p.i. and in two out of the three NCs included in the assay, yet no infectious virus was detected in these samples (Fig. 4).

### CHIKV viral protein synthesis in infected *ex vivo* guts

**Fig 5:**
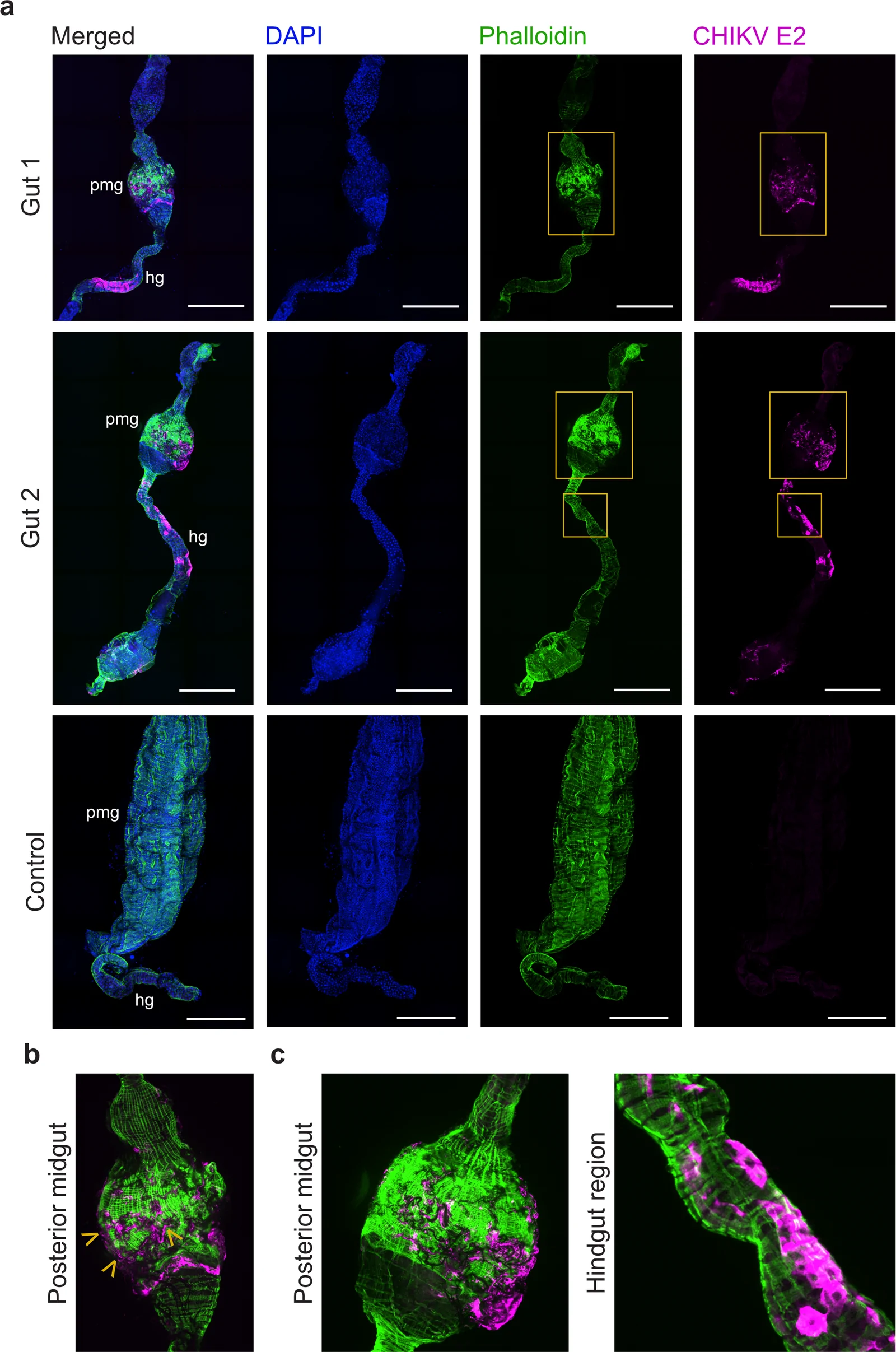
Detection of CHIKV protein synthesis in infected *ex vivo* gut cultures. (**a**) Magnification, 25×. Overlay of blue, green, and magenta filter imaging are shown for two infected gut organs and one NC gut. Imaging of each individual channel as follows: in blue, DAPI-stained cell nuclei; in green, actin filaments stained with phalloidin; and in magenta, E2 viral protein. No E2 expression was detected in the NC midguts. The scale bar in panels, represented by the white line, corresponds to 400 µm. pmg: posterior midgut. hg: hindgut. (**b**) Close-up panel showing infection foci in the posterior midgut region corresponding to the yellow squares indicated in (**a**) for “Gut 1.” Arrowheads indicate E2 expression localized in tracheal tubes of the midgut. (**c**) Close-up panel showing infection foci in the posterior midgut and hindgut region corresponding to the yellow squares indicated in (**a**) for “Gut 2.”

Previous infection experiments indicated the susceptibility of the *ex vivo* guts to infection with several arboviruses. To further corroborate these results, the CHIKV E2 glycoprotein was visualized in CHIKV-infected guts at day 3 p.i. by immunostaining. Specific staining for this viral protein could be observed in the infected guts, while no signal was observed in the NC (fixed guts that followed the same infection protocol; Fig. 5a). At day 3 p.i., E2 protein synthesis was mainly detected and spread along the posterior midgut region (Fig. 5b and c), with some infection foci located in the hindgut region (Fig. 5c). The E2 signal was also present in the tracheal tubes that remained attached to the midgut after dissection (Fig. 5b, arrowheads).

### Infection of *ex vivo* cultured guts with an mCherry-expressing DENV-2

**Fig 6:**
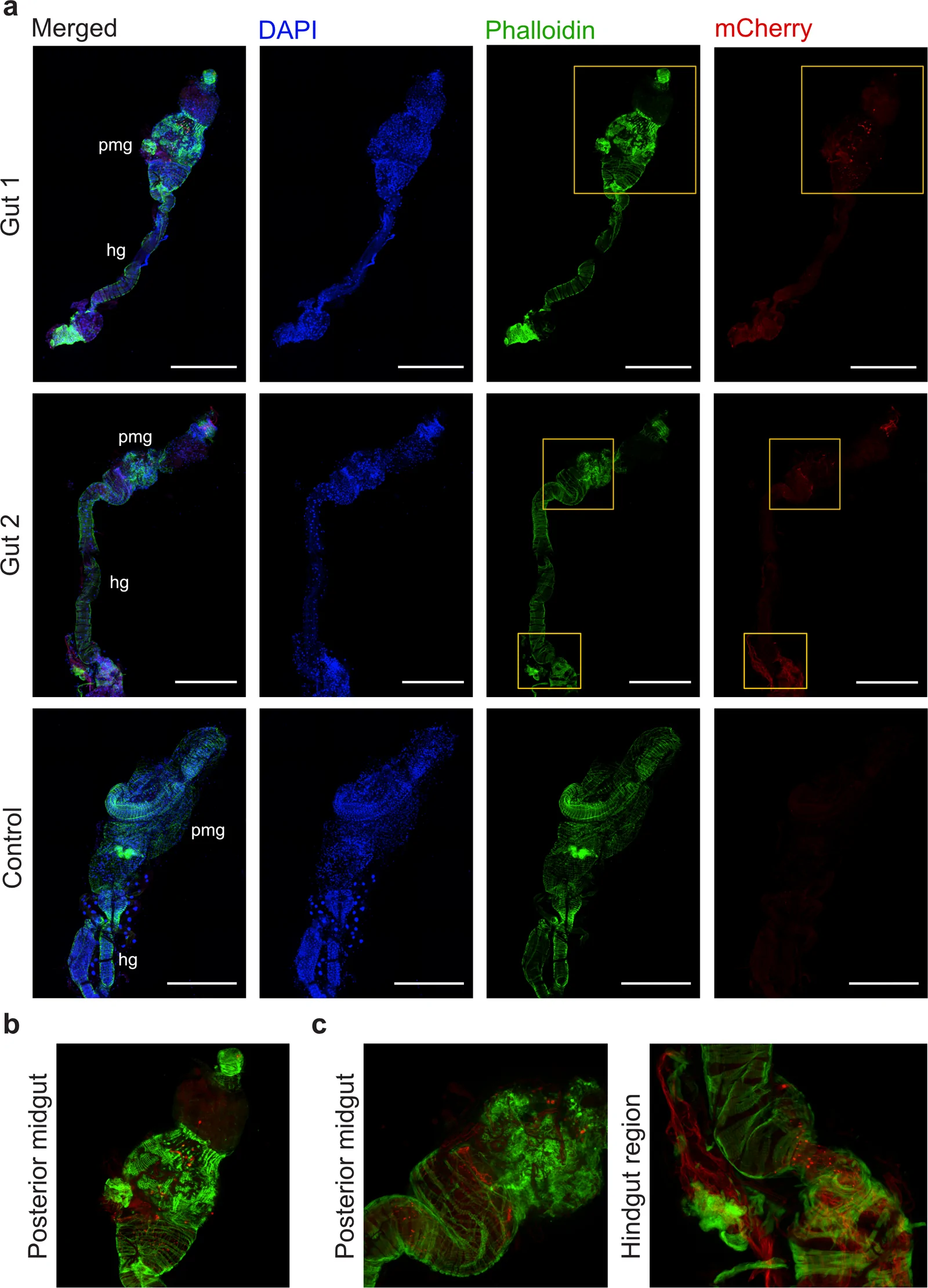
Replication of DENV-2-expressing mCherry in the *ex vivo* cultured guts. (**a**) Magnification, 25×. Confocal microscopy displays the DENV-2 infection in the *ex vivo* guts at day 7 p.i., as seen by the mCherry (red) signal. Overlay of blue, green, and red filter imaging are shown for two gut organs and one NC midgut. Imaging of each individual channel as follows: in blue, DAPI-stained cell nuclei; in green, actin filaments stained with phalloidin; and in red, mCherry signal. No mCherry expression was detected in fixed or mock-infected guts. The scale bar in panels, represented by the white line, corresponds to 400 µm. pmg: posterior midgut. hg: hindgut. (**b**) Close-up panel showing several infection foci in the posterior midgut region corresponding to the yellow squares indicated in (**a**) for “Gut 1.” (**c**) Close-up panel showing infection foci in the posterior midgut and hindgut region corresponding to the yellow squares indicated in (**a**) for “Gut 2.”

To follow the progression of virus infection in the guts by imaging, cultured mosquito guts were infected with DENV-2 expressing the red fluorescent protein mCherry (DV2/mCherry (18)) and imaged using the FLoid Cell Imaging Station (Life Technologies) at selected time points p.i.. The mCherry signal was used as a proxy for infection. DENV-2 mCherry infection was observed initially at day 3 p.i. as single or few foci in the posterior midgut region (Supplementary material, Fig. S3a). Over time, these focal infection points increased in number and spread to neighboring areas, primarily along the posterior region of the midgut and the hindgut (Supplementary material, Fig. S3b-e). No mCherry expression was observed in the NC or mock-infected guts at day 7 p.i. (Fig. 6a). Following 7 d of infection, the mCherry signal had spread mostly along the posterior midgut region, forming infection foci comprising multiple cells (Fig. 6b and c). Moreover, some infected cells were found in the tracheal tubes that remained in the midgut tissue after dissection (Supplementary material, Fig. S3h).

### Arbovirus replication is reduced upon treatment with antiviral drugs in gut cultures

**Fig 7:**
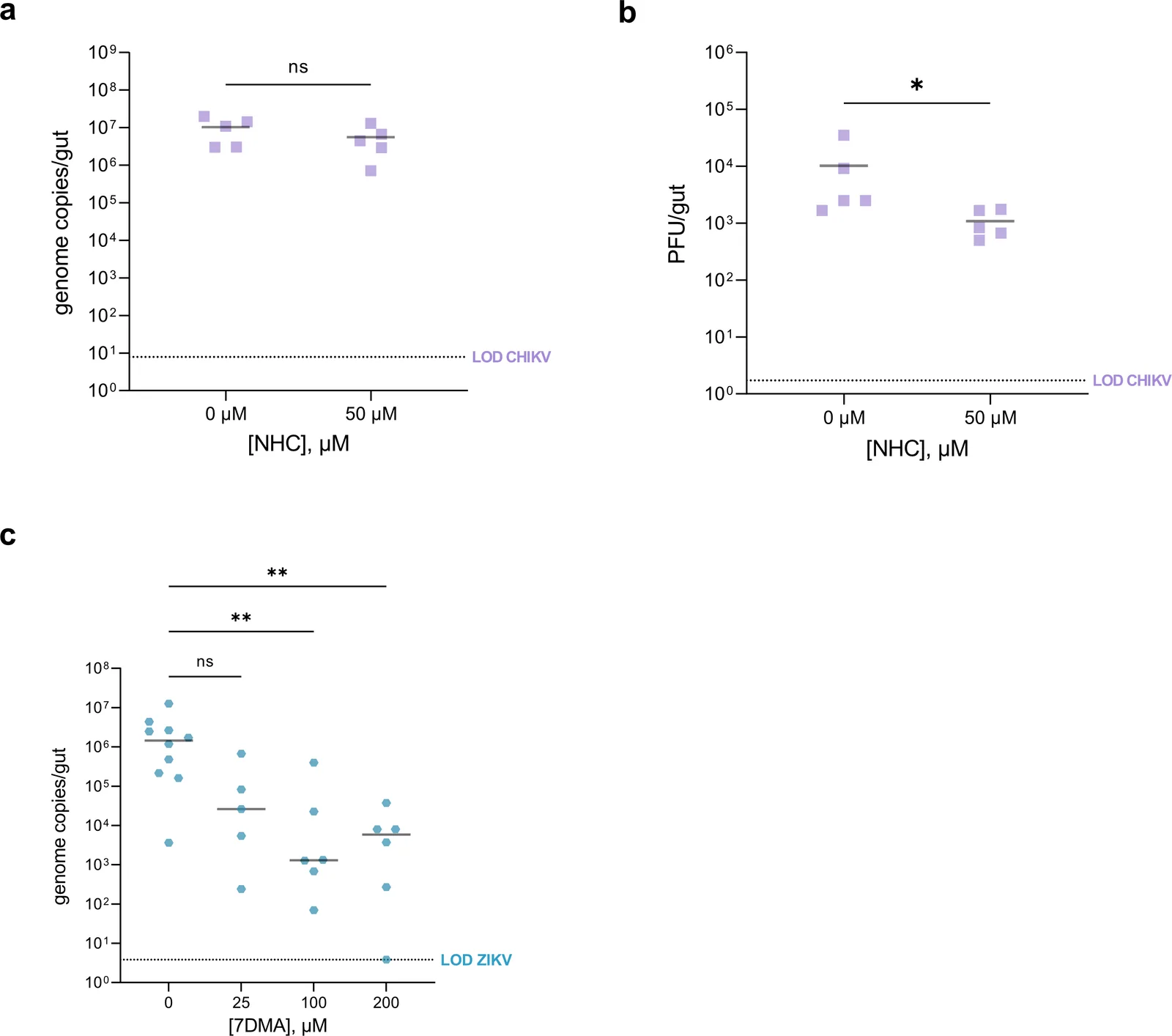
Antiviral activity of NHC and 7-deaza-2′-C-methyladenosine (7DMA) in the *ex vivo* cultured mosquito guts. (**a**) CHIKV RNA loads in the mosquito guts were quantified at day 3 p.i. by qRT-PCR. (**b**) Infectious virus loads were quantified at day 3 p.i. by plaque assay. Statistical significance was assessed with a Mann–Whitney test. Significantly different values are indicated by asterisks: *, *P* = 0.0238. (**c**) ZIKV RNA levels in the mosquito guts were quantified at day 7 p.i. by qRT-PCR. Statistical significance was assessed with the Kruskal–Wallis test. Significantly different values are indicated by asterisks: **, *P* = 0.0079. ns: not significant. Each dot represents an individual gut. The black line represents the median value. LOD: Limit of detection of the corresponding assay.

We next assessed whether the *ex vivo* mosquito guts could be used to evaluate the antiviral activity of inhibitors against arboviruses. To this end, the antiviral activity of β-d-N^4^-hydroxycytidine, also known as EIDD-1931 or NHC, was tested against CHIKV at a concentration of 50 µM (Fig. 7a). At day 3 p.i., no difference in the CHIKV RNA levels was observed between the untreated and NHC-treated groups. In contrast, NHC significantly reduced the infectious virus levels by one log (mean values, control: 1.0 × 10^4^; 50 µM NHC: 1.1 × 10^3^ PFU/gut). NHC had no effect on the viability of the *ex vivo* cultured guts as demonstrated by an ATP-based viability assay (Supplementary material, Fig. S4).

As the *ex vivo* cultured guts also supported flavivirus infection, the viral polymerase inhibitor 7-Deaza-2′-C-methyladenosine (7-DMA) was tested against ZIKV. Viral RNA levels were reduced for all treated guts (25, 100, and 200 µM of 7-DMA) compared to the untreated group at day 7 p.i. (Fig. 7b). However, only the groups treated with 7-DMA at 100 and 200 µM showed a significant difference compared to the untreated group, with 1.5 log and 2.4 log reductions (virus control: 2.6 × 10^6^; 100 µM: 7.0 × 10^4^; and 200 µM: 9.5 × 10^3^ mean genome copies/gut), respectively. 7-DMA did not exert toxic effects on the *ex vivo* cultured guts (Supplementary material, Fig. S4).

## DISCUSSION

The study of arbovirus infection in the mosquito vector relies mainly on a combination of *in vitro* and *in vivo* approaches, which has yielded great progress in the knowledge regarding arbovirus biology, virus-vector interactions, and vector competence. While convenient and handy, *in vitro* cell culture systems have the shortcoming of not being as biologically relevant as mosquito infection models, aside from the limited selection of mosquito cell lines available. On the other hand, working with living infected mosquitoes grants useful information when studying virus–vector interactions or vector competence for a specific virus, but such *in vivo* models are not always accessible and require the implementation of cumbersome safety measures ensuring adequate containment while working with BSL2/3 pathogens (19). *Ex vivo* organ culture methods have not yet been described in-depth for the study of MBVs. Hence, in this study, we have established an *ex vivo* mosquito gut model using dissected guts from *Ae. aegypti* mosquitoes. When cultured *ex vivo,* the guts displayed peristaltic contractions, mainly observed as waves along the hindgut, which is a frequent occurrence for this type of tissue (20). Here, we used this visual feature as a proxy for viability, paired with a custom script for gut motility in zebrafish (21) to measure the peristaltic period (time between contractions) in the *ex vivo* cultured guts, which remained stable for 7 d.p.d. This observation was further corroborated when measuring the metabolic activity of the dissected guts over time in culture with PrestoBlue (PB). This resazurin-based reagent has been successfully used to assess cell viability and cytotoxicity in both 2D cell monolayers and 3D cellular interfaces (including organ explants) (22 – 25). Here, we found that only after 7 h of incubation of the *ex vivo* guts (using multiple gut organs per well) in the presence of the PB reagent yielded a colorimetric change. The PB assay is based on the cellular reduction in resazurin. Only viable cells are able to reduce resazurin in cellular respiration, resulting in a colorimetric change from blue to red [conversion of resazurin (nonfluorescent form, blue) into resorufin (fluorescent form, red)] (23).

The *ex vivo* cultured mosquito guts supported infection with four different arboviruses from two virus families. Both viral RNA and infectious virus particles were detected in the mosquito gut organs following a progressive increase over time, which also confirmed the viability of the tissue. These replication kinetics results indicated the preservation of susceptible tissue in an *ex vivo* culture setup and, more specifically, the presence of midgut cells that could be infected by arboviruses, despite the unusual route of infection (through incubation with virus inoculum) that differs from what occurs when mosquitoes are naturally infected via blood feeding. When the midgut is infected via blood feeding, the virus reaches the lumen first and infects and replicates directly in the midgut epithelial cells. Once infection is established, viruses will cross through the basal lamina and disseminate into the hemolymph. In the *ex vivo* gut model, the virus has a direct contact with the basal lamina first (instead of the midgut epithelial cells), which could explain the less efficient infection and lower viral yields observed for some viruses in this study. Furthermore, as we have detected CHIKV infection localized in midgut-associated tracheae, we hypothesize that arbovirus infection and replication in the *ex vivo* gut could be facilitated by the tracheae present in the tissue. As such, infection could start in a tracheal cell and further infect muscles, consequently reaching the gut epithelium.

With *Ae. aegypti* being the main vector for both DENV-2 and ZIKV, it was unexpected that infectious virus titers for both flaviviruses detected in the *ex vivo* guts did not reach higher levels than what was inoculated (1 × 10^4^ PFU/mL), in contrast to viral RNA levels. Kinetics of DENV replication in mosquitoes have reported a steady increase in infectious virus until 8 d.p.i., after which it normally declined without affecting viral RNA levels (26, 27). Such results could explain the discrepancy seen between flavivirus RNA and infectious levels. More importantly, it has been described that within 2 h of infection with DENV or ZIKV (both *in vivo* and *ex vivo*), there is a rapid induction of apoptosis in the *Ae. aegypti* midgut epithelium in an attempt of the host to control flavivirus infection (28). Nonetheless, this process might cause tissue damage, which correlates with the high midgut cell turnover rate in mosquitoes during a bloodmeal digestion (29). As such, we cannot disregard that the *ex vivo* guts in our experiments undergo the same process during DENV or ZIKV infection, consequently damaging the tissue and thus limiting flaviviral replication. This hypothesis may also apply to *ex vivo* guts of *Cx. pipiens* mosquitoes, as a comparable replication pattern can be observed when assessing USUV (flavivirus) infection at day 7 p.i. Further addition of an apoptosis inhibitor to the virus inoculum used to infect the *ex vivo* guts might elucidate whether rapid induction of apoptosis in the midgut epithelium is indeed a limiting factor for flavivirus replication in this setup.

Active virus replication was confirmed for CHIKV through an immunofluorescence assay. Envelope (E2) protein synthesis was localized in the posterior midgut region, where it would also occur when the mosquito ingests a CHIKV-infectious bloodmeal (30), regardless of the infection method employed with the *ex vivo* cultured guts. Moreover, CHIKV infection of tracheal tubes in the tissue could be observed, consistent with *in vivo* reports (30). Similar results were obtained when analyzing the progression of DENV2/mCherry infection in the *ex vivo* guts. Previously, DENV-2 infection in *Ae. aegypti* (Chetumal strain) midguts was reported to start with individual infected epithelial cells detected as early as 2 d.p.i., slowly progressing to infection foci consisting of multiple cells until the whole midgut organ was infected at 7–10 d.p.i (26). In agreement with this, a less strong but akin infection pattern was observed in DENV2/mCherry-infected guts, with few foci of several infected cells by 7 d.p.i. Together, these data show that arbovirus infection occurred at considerable levels in *ex vivo* cultured guts and therefore it could be used to study other aspects of arbovirus infection and facilitate the collection of preliminary data before experimenting with mosquitoes *in vivo*.

The use of antiviral compounds to inhibit virus infection in the mosquito vector is an innovative concept that might reduce or block arbovirus transmission from mosquitoes to humans. This idea involves antiviral molecules being ingested by adult mosquitoes when they take a bloodmeal on a mammalian host undergoing antiviral treatment. As the mosquito midgut is the entry point and key replication site for MBVs, studying the effect of antiviral compounds in the midgut tissue would be of great interest. For this purpose, the antiviral activity of two inhibitors was assessed in the *ex vivo* mosquito guts. NHC is a nucleoside analog that has been characterized as a potent antiviral drug against alphaviruses *in vitro*, including CHIKV and Venezuelan equine encephalitis virus (31, 32). Various assays point to the compound acting as a pyrimidine analog that may target the viral polymerase domain of nsP4, provoking chain termination (33). In addition, NHC also induces a high level of mutations in virus-specific RNAs, resulting in lethal mutagenesis (34). Consistent with these findings, CHIKV RNA levels were not reduced in NHC-treated guts, but there was a marked decrease in virus infectivity. The viral polymerase inhibitor 7DMA has shown potent anti-ZIKV activity *in vitro* and delayed disease progression in mice (35). In the *ex vivo* mosquito guts, 7DMA significantly reduced ZIKV RNA loads. Although the RNA levels presented some variability among the ZIKV-infected guts, the inhibitory effect of 7DMA in the *ex vivo* mosquito guts was significant and followed a dose–response relationship. These results indicate that *ex vivo* gut cultures could be used to rationally select potential arbovirus-blocking molecules to be tested in living mosquitoes at a later stage.

In summary, we have established a long-term *ex vivo* mosquito gut culture. To support this model, we have (a) assessed the viability of the *ex vivo* cultured guts, (b) determined the replication kinetics of two alphaviruses (RRV and CHIKV) and two flaviviruses (DENV-2 and ZIKV), (c) detected viral protein synthesis and followed live arbovirus infection in infected guts, (d) demonstrated that the *ex vivo* protocol can be translated to other mosquito genera, and, last, (e) evaluated the antiviral activity of two inhibitors against CHIKV and ZIKV. Altogether, we have provided a reference for the use of *ex vivo* mosquito guts as a tool to study arbovirus infection and related processes while offering groundwork for developing other *ex vivo* mosquito organ cultures that can potentially provide insightful data, like salivary glands.

## MATERIALS AND METHODS

### Cells

#### Mammalian cells

African green monkey kidney cells (Vero cells, ATCC CCL-81) and Vero E6 cells (ATCC CRL-1586) were cultured in minimum essential medium (1×) enriched with 10% fetal bovine serum (FBS), 1% sodium bicarbonate, 1% L-glutamine, and 1% nonessential amino acids (NEAA). Baby hamster kidney cells (BHK, ATCC CCL-10) were maintained in Dulbecco’s Modified Eagle’s Medium containing 10% FBS, 1% sodium bicarbonate, and 1% L-glutamine. Mammalian cell cultures were incubated at 37°C, with 5% CO_2_.

#### Mosquito cells

*Ae. albopictus* larval cells (C6/36, obtained from ATCC, CRL-1660) were maintained in Leibovitz’s L-15 medium containing 10% FBS, 1% penicillin–streptomycin (Pen-Strep), 1% NEAA, and 1% HEPES buffer. Mosquito-derived cell lines were incubated at 28°C, without CO_2_.

For cell culture assays containing virus or virus-infected material, the concentration of FBS in the medium was reduced to 2%, for both mammalian and mosquito cells. All cell culture media and supplements were obtained from Gibco, ThermoFisher Scientific (Aalst, Belgium).

### Viruses

#### Flaviviruses

DENV serotype 2 (DENV-2/TH/1974, isolated in 1974 from human serum collected in Bangkok, Thailand, GenBank MK268692.1) was kindly provided by Prof. A. Failloux (Institut Pasteur, Paris, France) (36); ZIKV (SL1602, Suriname strain, GenBank KY348640.1) was acquired via the EVAg consortium (https://www.european-virus-archive.com). The infectious clone DENV-2 pDVWS601 used for the construction of the DENV-2 reporter virus expressing the red fluorescent protein mCherry (DV2/mCherry, New Guinea C strain, NGC, GenBank AF038403.1) was kindly provided by Prof. Andrew Davidson (University of Bristol, Bristol, UK) (18).

#### Alphaviruses

RRV was received from the National Collection of Pathogenic Viruses (UK; catalog number 0005281 v), and CHIKV (Indian Ocean strain 899, GenBank FJ959103.1) was generously provided by Prof. Drosten (University of Bonn, Bonn, Germany) (37).

Virus stocks were prepared by passaging the isolates on Vero (for CHIKV and USUV) or C6/36 cells (for ZIKV, DENV-2, and RRV). Viral titers of the stocks were determined via plaque assay or end point titration on Vero or BHK cells.

### Compounds

7-DMA was purchased from Carbosynth (Berkshire, UK) and dissolved in dimethylsulfoxide (DMSO). EIDD-1931 or NHC was purchased from MedChemExpress (Monmouth Junction, NJ, USA) and dissolved in DMSO.

#### *Ae. aegypti* rearing

*Ae. aegypti* Paea (Papeete, Tahiti, collected in 1994) were obtained via the Infavec2 consortium and reared continuously in the laboratory (36). Adult mosquitoes were regularly blood-fed to produce eggs and maintain the mosquito colony. In brief, female mosquitoes were offered freshly collected rabbit blood using an artificial membrane feeding system (Hemotek, UK). After feeding, blood-engorged females were selected and placed in a cage. A water-filled glass reservoir with a paper lining the wall (for the females to lay their eggs) was positioned inside the cage. After a week, the egg-containing paper was collected, left to dry, and further stored in a sealed bag. These eggs were used to rear mosquitoes for the experiments presented in this study. For each rearing, eggs were hatched in dechlorinated tap water. After hatching, groups of ±400 larvae were transferred into trays containing 3 L of dechlorinated tap water and fed every day with a yeast tablet (Gayelord Hauser, Saint-Genis-Laval, France) until the pupae stage. Pupae were placed in small plastic containers inside cardboard cups for their emergence. Adult mosquitoes were supplied with cotton balls soaked in a 10% sucrose solution supplemented with 100 U/mL and 100 µg/mL of Pen-Strep. Cardboard cups containing adults were maintained at 28 ± 1°C with a light/dark cycle of 16/8 h and 80% relative humidity.

#### *Cx. pipiens* rearing

*Cx. pipiens* biotype *pipiens* were kindly provided by Prof. Sander Koenraadt (Wageningen University & Research, Wageningen, Netherlands) (17). Adult mosquitoes were regularly blood-fed to produce eggs and maintain the mosquito colony, in a similar manner as described above for *Ae. aegypti* mosquitoes. For *Cx. pipiens*, chicken blood was used for blood-feeding and the egg rafts produced by the female mosquitoes were collected and immediately hatched as these eggs cannot be stored. For each rearing, eggs rafts were hatched in trays containing 2 L of Milli-Q water (Synergy UV, Merck, Germany). Larvae were fed continuously until the pupae stage with TetraMin baby fish food (Tetra, Spectrum Brands, Germany). Pupae were collected as described above for *Ae. aegypti*. Cardboard cups containing adults were maintained at 25 ± 1°C with a light/dark cycle of 16/8 h and 70% relative humidity.

### Mosquito gut dissection and *ex vivo* culture

Unfed, antibiotic-treated female mosquitoes (3–7 d old) were cold-anaesthetized and surface sterilized by soaking in 70% ethanol for 20 s followed by soaking in Dulbecco’s phosphate-buffered saline (PBS) for 20 s. Next, mosquitoes were dissected in PBS on a petri dish using the stereomicroscope (VisiScope, VWR). In brief, the gut was exposed by carefully pulling the second to last segment of the mosquito abdomen. The gut of the mosquito was excised including its three main compartments (the foregut, the midgut, and the hindgut) to keep the tissue of interest (midgut) from degradation, and tracheal tubes were removed as much as possible without damaging the tissue. Malpighian tubules were also removed carefully from the gut as the goal was to keep the main alimentary canal. Following dissection, mosquito guts were washed twice in gut medium, consisting of Leibovitz’s L-15 medium supplemented with 2% FBS, 100 U/mL of Pen-Strep, 50 µg/mL of kanamycin, and 0.25 µg/mL of amphotericin B (Sigma Aldrich, USA), and finally placed in a 96-well tissue culture plate with a clear bottom (PerkinElmer, USA) containing gut medium. One mosquito gut was placed in each testing well. Plates were maintained at 28°C, without CO_2_.

Of note, handling and optimization of the *ex vivo* mosquito guts culture are extensively described in Supplementary Appendix 1.

### Videography of gut peristalsis and analysis

*Ex vivo* cultured mosquito guts were recorded with the Leica DMi8 microscope to quantify the peristalsis observed *ex vivo*, specifically in the hindgut region. A drop of carboxymethyl cellulose (CMC) 0.8% diluted in Leibovitz’s L-15 medium was deposited on a microscope glass slide and one mosquito gut was soaked in the drop. Forceps were used to gently arrange the hindgut in a position suitable for analysis (horizontally positioned on the slide). Each hindgut was recorded for a total of 1 min 7 s (one frame/0.3 s) and further discarded. This procedure was repeated at several time points during incubation starting from day 0 (right after dissection) to assess the contractibility, and hence viability, of the *ex vivo* cultured guts over time.

For the analysis of the gut peristalsis, frames generated for each hindgut were processed in IgorPro (Wavemetrics, USA) using a custom script described for the measurement of zebrafish gut motility (21). This script quantifies the changes in pixel intensity in a designated area of analysis as it sequentially goes through all frames generated during the video recording. As output, the software indicates individual peristaltic periodicity (seconds in between contractions) per point of evaluation and overall averages of peristaltic periodicity for each midgut analyzed. These outputs were annotated manually to check for artifacts that could be generated by debris or tracheal tubes.

Serotonin hydrochloride (Sigma Aldrich, USA) and atropine sulfate salt monohydrate (Sigma Aldrich, USA) were used to test the responsiveness of the mosquito gut tissue. Guts were incubated with serotonin (20 µM), atropine (20 µM), or gut medium for 1 h, after which they were video recorded for further analysis.

### PrestoBlue (PB) assay

The metabolic activity of the dissected guts was further assessed using a resazurin salt-based cell viability reagent PrestoBlue (Invitrogen, USA). For these experiments, three dissected guts were placed per well in a 96-well tissue culture plate. Each trio of guts was considered a biological replicate. Using more than one gut per well was necessary to ensure both a robust readout and a relatively rapid change of color in the testing medium. Guts fixed in 4% PFA (Sigma Aldrich, USA) were included in the assay as negative controls (NC). No organ (NO) controls were composed of wells without gut organs. In addition, one tip of the forceps used for dissection was dipped inside each NO control well to simulate putting a gut organ inside, as with other wells, and account for any contamination carried by the dissection tools.

The live guts and the corresponding controls were tested with PB every day for a period of 10 d. In brief, the medium was carefully removed from the wells and replaced with 100 µL of a 1:10 dilution of PB prepared in gut medium (without phenol red). Next, the guts and their controls were incubated at 28°C without CO_2_ for 7 h. Once the incubation time was completed, the PB-containing medium was transferred to a 96-well tissue culture plate and fluorescence intensity was measured at wavelengths 560 nm excitation and 590 nm emission with a Spark Multimode Microplate Reader (Tecan Trading AG, Switzerland). New gut medium was added to wells containing guts and they were returned to the incubator (28°C, no CO_2_) until the next time point assessment. Two independent assays were carried out with at least three biological replicates per time point.

### Infection and antiviral assays in *ex vivo* mosquito guts

Mosquito guts were dissected as described above and placed into a 96-well tissue culture plate filled with gut medium. After removing the medium, a total of 1 × 10^4^ PFU/mL of the virus was added to each well. Of note, inocula of 1 × 10^5^ and 1 × 10^6^ PFU/mL were used for DENV-2 and USUV, respectively. Negative controls consisted of dissected guts fixed in 4% PFA for 30 min. Guts were incubated for 2 h at 28°C, without CO_2_. The virus inoculum was carefully removed, and guts were washed two times with gut medium. Fresh medium was finally added to all wells for incubation at 28°C, without CO_2_.

To test the activity of antiviral drugs in the *ex vivo* mosquito guts, compound dilutions were prepared in gut medium and added to the wells containing guts to be treated, after which they were infected with 1 × 10^4-5^ PFU/mL of virus. Virus control guts were incubated with the virus inoculum only. Following 2 h of incubation at 28°C, without CO_2_, the guts were washed twice before adding gut medium alone (for virus control guts) or containing compound (for treated guts). Guts were returned to the incubator at 28°C, without CO_2_.

Incubation time for alphavirus-infected guts was 3 d, while flavivirus-infected guts were maintained for 7 d. Guts were collected at several time points after infection for further analysis.

### Determination of viral RNA levels and detection of infectious virus replication

Collected guts were homogenized individually in 250 µL of PBS using bead disruption (2.8 mm beads, Precellys). The gut homogenate was filtered using 0.8 µm MINI column filters (Sartorius, Germany) to remove debris, bacteria, and fungi. The filtered homogenate was used for further viral RNA isolation and qRT-PCR to determine viral RNA levels, and plaque assay to assess infectious virus particles.

Viral RNA isolation was performed with the NucleoSpin RNA Virus kit (Macherey-Nagel, Germany) following the manufacturer’s protocol. The sequences of primers and probes used for each virus are compiled in Table 1. One-Step, quantitative RT-PCR was performed for CHIKV and ZIKV in a total volume of 25 µL, consisting of 13.94 µL of RNase-free water (Promega, USA), 6.25 µL of master mix (Eurogentec, Belgium), 0.375 µL of each forward and reverse primer (to a final concentration for each primer: 150 nM [CHIKV and USUV]; 900 nM [ZIKV]), 1 µL of probe (to a final concentration of 400 nM [CHIKV and USUV]; 200 nM [ZIKV]), 0.0625 µL of reverse transcriptase (Eurogentec, Belgium), and 3 µL of RNA sample. For RRV, the reaction mixture was prepared in a total volume of 20 µL, containing 5.2 µL of RNase-free water, 10 µL of SYBR Green master mix (BioRad, USA), 1 µL of each forward and reverse primer (to a final concentration of 125 nM for each primer), 0.3 µL of reverse transcriptase (BioRad, USA), and 4 µL of RNA sample. For DENV-2, the reaction mixture was prepared to a final volume of 20 µL, consisting of 3 µL of RNase-free water, 10 µL of SYBR Green master mix (BioRad, USA), 0.3 µL of each forward and reverse primer (to a final concentration of 900 nM for each primer), 0.4 µL of reverse transcriptase (BioRad, USA) and 6 µL of RNA sample.

**TABLE 1:** Sequences of primers and probes for qRT-PCR used in this study

Virus [target protein]	Forward primer (5’ → 3’)	Reverse primer (5’ → 3’)	Probe (5’ → 3’)	Reference
Ross River virus [E2]	TACAAGCACGACCCATTGCCG	GATAGTCCTGCCGCCTGCTGT	N/A	(38)
Chikungunya virus [nsP1]	CCGACTCAACCATCCTGGAT	GGCAGACGCAGTGGTACTTCCT	FAM-TCCGACATCATCCTCCTTGCTGGC-TAMRA	(39)
Zika virus [E]	CCGCTGCCCAACACAAG	CCACTAACGTTCTTTTGCAGACAT	FAM-AGCCTACCT-ZEN-TGACAAGCAATCAGACACTCAA-IABkFQ	(40)
Dengue virus serotype 2 [NS3]	CAGATCGGAGCTGGAGTTTAC	TTTTGACGTCCGCCCATGAA	N/A	*In-house*
Usutu virus [NS5]	AAAAATGTACGCGGATGACACA	TTTGGCCTCGTTGTCAAGATC	FAM-TGGGACACCCGGATACCAGAG-TAMRA	(41)

The qRT-PCR assays were performed using the QuantStudio 5 Real-Time PCR System (ThermoFisher Scientific, USA) with the following cycling conditions for CHIKV, ZIKV, and USUV: 30 min at 48°C, 10 min at 95°C, followed by 40 cycles of 15 s at 95°C and 1 min at 60°C (55°C for USUV). Cycling conditions for RRV were as follows: 10 min at 50°C, 3 min at 95°C, followed by 40 cycles of 15 s at 95°C and 30 s at 60°C. For DENV-2, the following cycling conditions were used: 30 min at 48°C, 3 min at 95°C, followed by 40 cycles of 15 s at 95°C and 1 min at 62°C.

For absolute quantification, standard curves were generated each run using 10-fold serial dilutions of cDNA of CHIKV-nsP1, viral RNA isolated from RRV, and DENV-2 Bangkok virus stocks or synthesized gBlocks gene fragments (Integrated DNA Technologies, USA) for ZIKV and USUV.

Plaque assays were performed to quantify infectious virus particles from infected gut samples. Mammalian cells (Vero cells for RRV and CHIKV; BHK cells for ZIKV, DENV-2, and USUV) were seeded at a density of 2.5 × 10^5^ cells per well in 24-well tissue culture plates. One-day confluent monolayers of cells were inoculated with serial 10-fold dilutions of gut samples, dilutions were prepared in 2% assay medium, and the inoculum was allowed to infect the cells for 2 h at 37°C, with 5% CO_2_. A negative control consisting of 2% assay medium used for preparing the dilutions was included in all plaque assays. The inoculum was removed and replaced with CMC 0.8% overlay diluted in RPMI medium (supplemented with 1% HEPES, 1% sodium bicarbonate, 1% L-glutamine, 1% Pen-Strep, and 2% FBS). Infected cells were incubated for 3 (*Alphaviruses*) or 7 (*Flaviviruses*) days. After incubation, 1 mL of 4% PFA was added to each well on top of the overlay medium and allowed for fixation for 1 h, after which the overlay mixture was discarded, and the wells were carefully washed with water. The culture plates were allowed to air dry, and then the wells were stained with 1% crystal violet solution (Sigma Aldrich, USA). Plaques were counted and titer of each sample was calculated.

### Immunofluorescence staining of CHIKV viral proteins and imaging

Mosquito guts were dissected and infected with CHIKV as described in the sections "Mosquito gut dissection and *ex vivo* culture" and "Infection and antiviral assays in *ex vivo* mosquito guts", respectively. At day 3 p.i., guts were fixed with 4% PFA for 1 h at room temperature. Fixed guts were transferred to a µ-slide 18-well (Ibidi GmbH, Germany) plate and rinsed with PBS five times, for 5 min each time. Guts were then saturated with PBS-T [0.1% Triton X-100 (Sigma Aldrich, USA) and 1% bovine serum albumin (BSA, Sigma Aldrich, USA) in PBS 1×) for 2 h at room temperature. Primary antibody anti-E2 protein [Chk265] (Absolute Antibody, Wilton, UK) was diluted in PBS-T (1:500) and added to the fixed guts for overnight incubation at 4°C. After incubation, guts were rinsed five times with PBS-T, for 5–10 min each rinse, followed by incubation with Alexa Fluor 594 donkey anti-rabbit secondary antibody (ThermoFisher Scientific, USA, A-21207, diluted in PBS-T, 1:500) for 2 h at room temperature. Guts were rinsed five times with PBS-T, for 5–10 min each rinse. Phalloidin (diluted in 1% BSA in PBS 1× solution, final concentration 100 nM) incubation followed for 10 min, after which DAPI (diluted in PBS 1×, final concentration 100 nM) was added on top of the phalloidin solution and allowed to incubate for 10 min. Finally, the phalloidin/DAPI solution was removed, and guts were rinsed with PBS. Gut samples were mounted in microscope slides using Glycergel mounting medium (Agilent, USA) and allowed to dry before imaging with a Leica DMi8 microscope (Leica Microsystems, Germany) and the Andor Dragonfly Confocal Spinning Disk microscope (Oxford Instruments, UK). All incubation steps mentioned above were carried out on rotation, at 300 rpm.

### Infection of *ex vivo* cultured guts with DENV-2-expressing mCherry and imaging

Mosquito guts were dissected and infected with a DENV-2-expressing mCherry (18) as described in the sections "Mosquito gut dissection and *ex vivo* culture" and "Infection and antiviral assays in *ex vivo* mosquito guts", respectively, using an inoculum of 1 × 10^5^ PFU/mL. Negative controls and mock-infected guts were included. Mock-infected guts were incubated in gut medium instead of virus dilution. Guts were monitored every day during the incubation period and checked under the FLoid Cell Imaging Station (Life Technologies) for any signal that indicated mCherry expression. At day 7 p.i., the mosquito guts were fixed with 4% PFA for 1 h at room temperature. After fixation, guts followed the protocol described above for immunofluorescence staining, skipping the addition of any primary and secondary antibodies.

## SUPPLEMENTAL MATERIAL

Supplemental material spectrum.05195-22-s0001.docx Supplemental Appendix S1, Figures S1 to S7, and legend of Movie S1.

ASM does not own the copyrights to Supplemental Material that may be linked to, or accessed through, an article. The authors have granted ASM a non-exclusive, world-wide license to publish the Supplemental Material files. Please contact the corresponding author directly for reuse.
