# Supplementary material for: Ex vivo gut cultures of Aedes aegypti are efficiently infected by mosquito-borne alpha- and flaviviruses
Source: Microbiol Spectr. 2023 Aug 4;11(5):e05195-22. doi: 10.1128/spectrum.05195-22 (PMC10580962; doi:10.1128/spectrum.05195-22)
Supplement: Supplemental material — Supplemental Appendix S1, Figures S1 to S7, and legend of Movie S1. [file spectrum.05195-22-s0001.docx]

**Appendix 1. Handling and optimization of *ex vivo* mosquito guts**

Thorough optimization has been carried out to make the *ex vivo* mosquito gut model an accessible and convenient tool for the study of arboviruses in the mosquito vector.

Initially, mosquito guts were placed in the well of a 96-well tissue culture plate, in which they remained suspended in culture medium. However, certain procedures (washing steps) were challenging due to the small size of the guts and the high probability of pipetting out the tissue. Therefore, other support systems were tested to immobilize the midguts and facilitate their handling to overcome this limitation.

To this end, Matrigel® (Corning®) domes were deposited in 48-well tissue culture plates (Corning® Costar®), in which the guts were placed before the dome completely solidified. Matrigel™ provides structural support for cells because of its content of laminin, collagen IV, entactin, and heparin sulfate proteoglycan perlecan, among other factors (32), which are the main components of the mosquito midgut basal lamina secreted by epithelial cells (33). The support given by the hydrogel could be readily observed when embedding and culturing the midguts in Matrigel™. Microscopic observation of the *ex vivo* guts cultured in medium showed some variation in the midgut organ integrity. Some midguts preserved their shape with a minimal amount of degradation over time, whereas others lost their integrity and progressively became misshapen, which has been noted before when working with mosquito tissue cultures (20). Of note, the hindgut was the region less prone to degradation. Such occurrence was improved greatly when using Matrigel™, as the guts preserved their integrity better compared to the traditionally used culture medium. Therefore, the domes provided good support for the gut organ, as they still presented hindgut peristalsis while keeping the tissue immobilized in the center of the well. However, infection of guts kept in Matrigel domes by incubation with ZIKV was not successful. No viral RNA was detected in these guts after infection (data not shown), as opposed to the guts cultured traditionally (suspended in medium). Considering that the current route of infection diverges from a natural mosquito infection, the use of synthetic hydrogels could help to overcome such limitation while providing an adequate environment to culture the *ex vivo* mosquito guts. For instance, the guts embedded in a hydrogel are immobilized, which could allow the microinjection of virus directly into the midgut region. Whether such approach could resemble the natural infection pattern of the midgut in mosquitoes warrants further investigation.

Another method tested consisted of using a foam substrate insert to line up the bottom of each well of the 96-well tissue culture plate filled with medium, as it was shown to be successful for the *ex vivo* culture of tick organs (14). Of note, the same foam substrate reported for tick organs could not be purchased due to its commercial unavailability. After dissection, mosquito guts were laid into the sponge substrate and kept in culture. Unfortunately, the sponge substrate used had big pores that allowed the guts to pass through the foam to the bottom of the well and posed difficulties in the washing steps; therefore, this method was also discarded. Of note, infection by incubation with ZIKV was successful in the guts kept on foam substrate; however, traditionally culture guts reached higher ZIKV RNA loads as measured by qRT-PCR (Supplementary material, Fig. S5). Furthermore, no organ controls, consisting of foam substrate (no gut) that went along all infection steps, were implemented to measure any virus inoculum that could remain embedded in the substrate. These no organ controls were shown to retain a higher amount of the virus inoculum compared to the negative controls normally used (Paraformaldehyde [PFA] fixed guts suspended in medium).

Therefore, the culture method that provided the best outcome in terms of handling, tissue preservation, and permissiveness to arbovirus infection was to keep the mosquito guts suspended in culture medium in a 96-well tissue culture plate with clear bottom (PerkinElmer©, USA). To reduce the risk of pipetting out the guts during washing steps, a magnifying lamp was key to observe the guts with the naked eye and avoid such problem. Lastly, carboxymethyl cellulose (CMC) 0.8% diluted in Leibovitz’s L-15 medium was also employed and found to be suitable for long incubation periods (>3 days), as it provided a denser medium where the guts seemed to preserve their shape better over time. However, viral RNA levels detected in the guts incubated in CMC were significantly reduced at day 2 and 3 p.i. compared to traditionally *ex vivo* cultured guts (Supplementary material, Fig. S6).


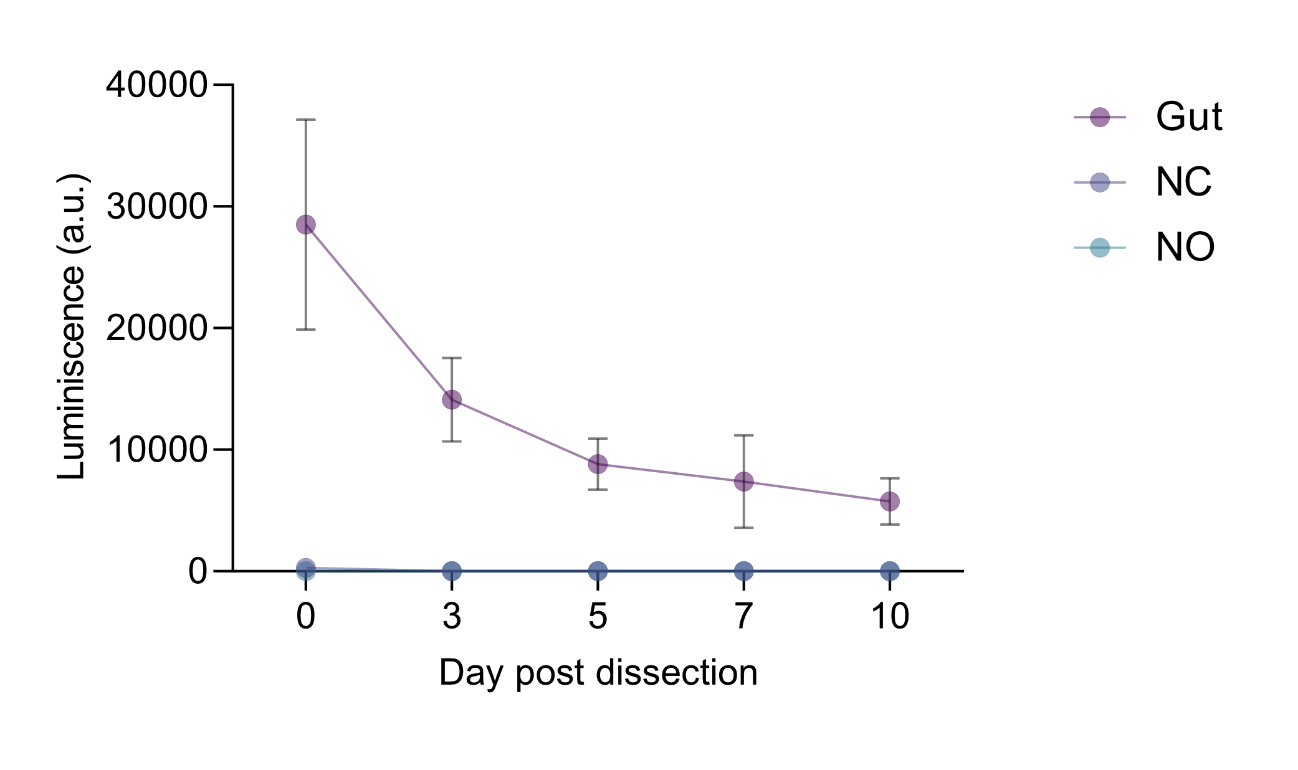


**Figure S1**. **Viability of the *ex vivo* cultured guts based on ATP measurements over 10 days.** Luminescence readouts for a period of 10 days for tested guts, no organ (NO), and negative controls (NC). Assays were performed with 5 biological replicates per time point, each replicate composed of 1 gut per well, along with its corresponding controls. Error bars represent the standard errors of the means per time point. The graph shows data from two independent assays.


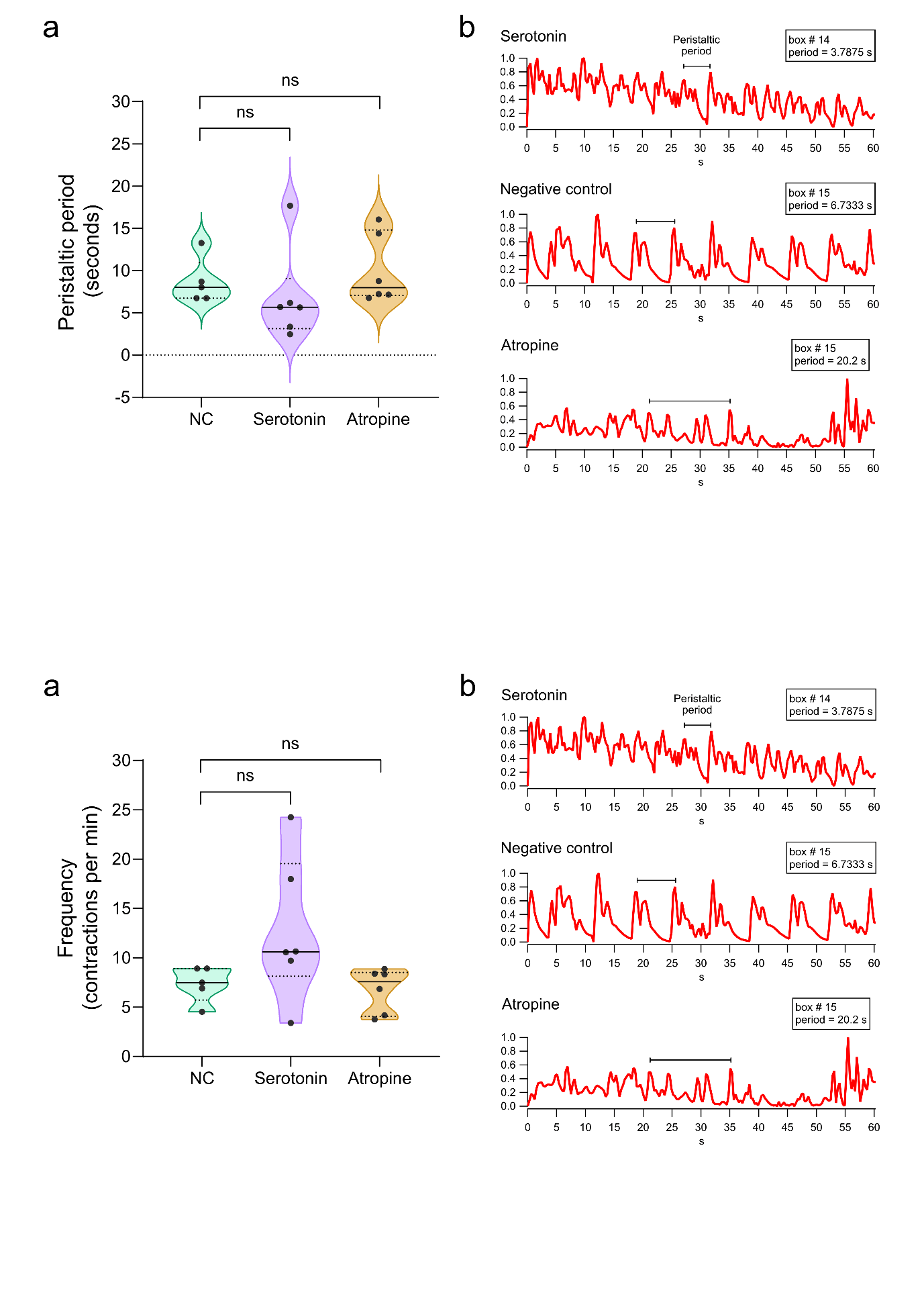


**Figure S2**. ***Ex vivo* cultured mosquito guts were not significantly affected by external incubation with serotonin or atropine.** **a**, Peristaltic period for each condition evaluated. NC stands for negative control, which represents the mock-exposed guts (untreated). Each point represents an individual mosquito gut. The line represents the median value. The experiment was performed once. **b**, Representative output analysis for one gut of each tested condition. The output corresponds to one region (box number) of the area of analysis (AOA) set for each gut. The peristaltic period in each output is representatively denoted as a black bar.


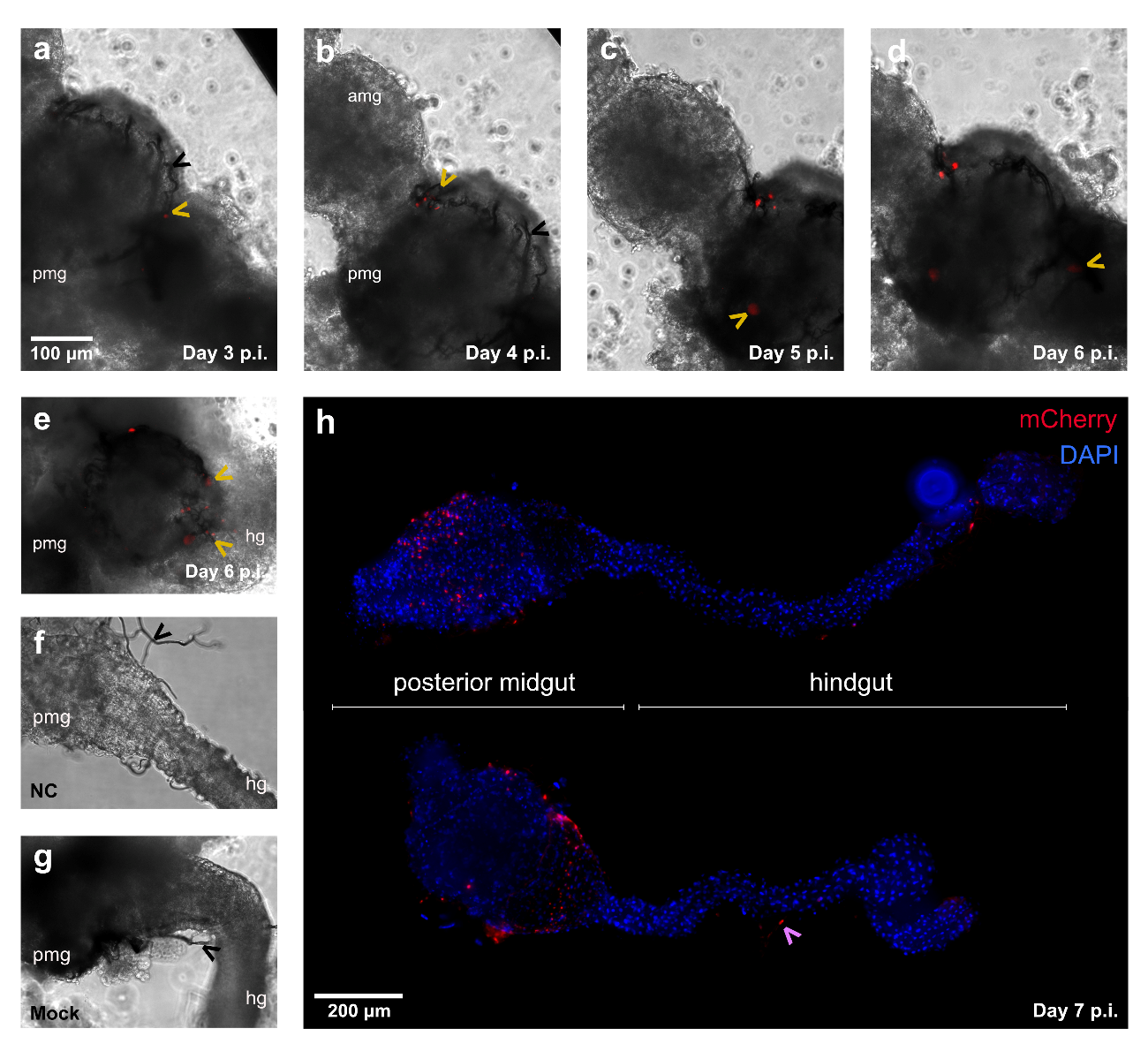


**Figure S3**. **Replication of DENV-2 expressing mCherry in the *ex vivo* cultured guts.** **a**, **b**, **c**, **d**, Magnification, 20X. Overlay of bright field and red filter imaging. Panels display the DENV-2 infection and spread in the guts as seen by the mCherry red signal on days 3 to 6 p.i. All four images correspond to the same organ imaged over the selected time points. The scale bar, represented by the white line, can be applied to panels a-g. **e**, mCherry expression in another biological replicate at day 6 p.i., showing several infection foci in the posterior midgut region. **f**, **g**, NC: negative control. No mCherry expression was detected in fixed or mock-infected guts. **h**, mCherry signal in DENV-2 infected gut, magnification, 10X. amg: anterior midgut, pmg: posterior midgut, hg: hindgut. Black arrows indicate the presence of tracheae. Yellow arrows indicate mCherry signal. Magenta arrow indicates mCherry signal present in tracheal tubes that remained in the hindgut.


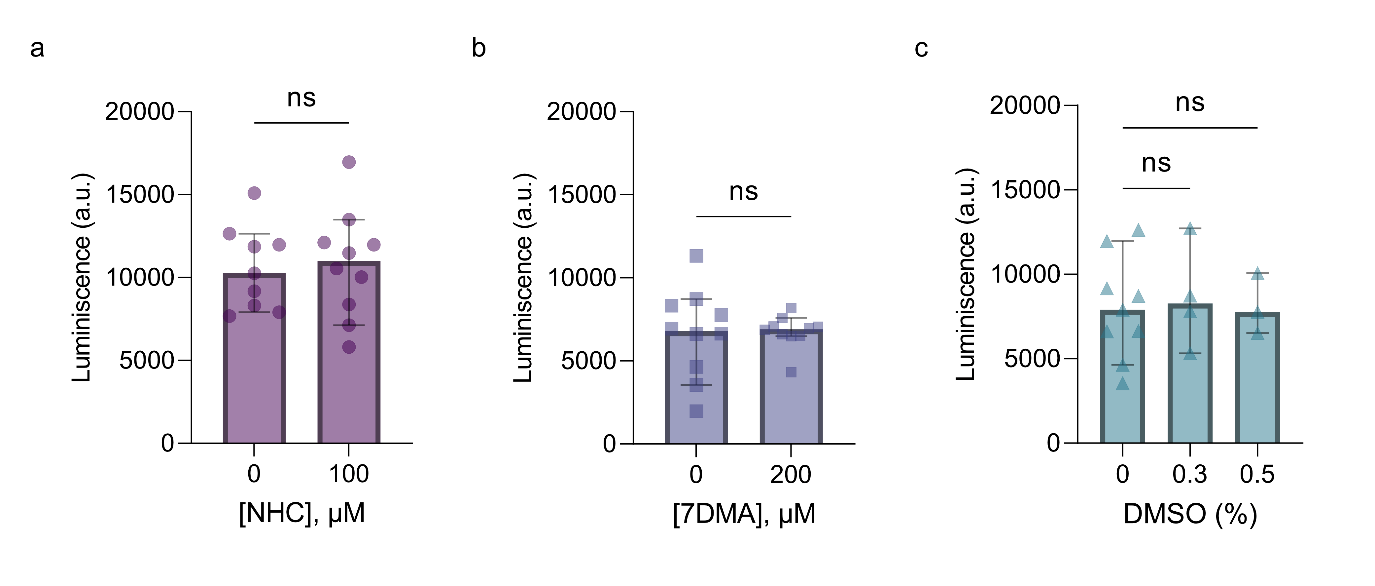


**Figure S4. Compound and solvent toxicity in the *ex vivo* guts.** The toxicity assays were performed using ATP as an indicator of viability (CellTiter-Glo® 3D). **a**, Luminescence readouts for guts incubated with 0 and 100 µM of NHC. **b**, Luminescence readouts for guts incubated with 0 and 200 µM of 7-DMA. **c**, Luminescence readouts for guts incubated with 0, 0.3, and 0.5 % of DMSO. For all conditions, statistical significance was evaluated with an unpaired t test. ns: not significant. Each symbol represents an individual gut organ. The height of the bar represents the median values with the 95 % confident interval superimposed.


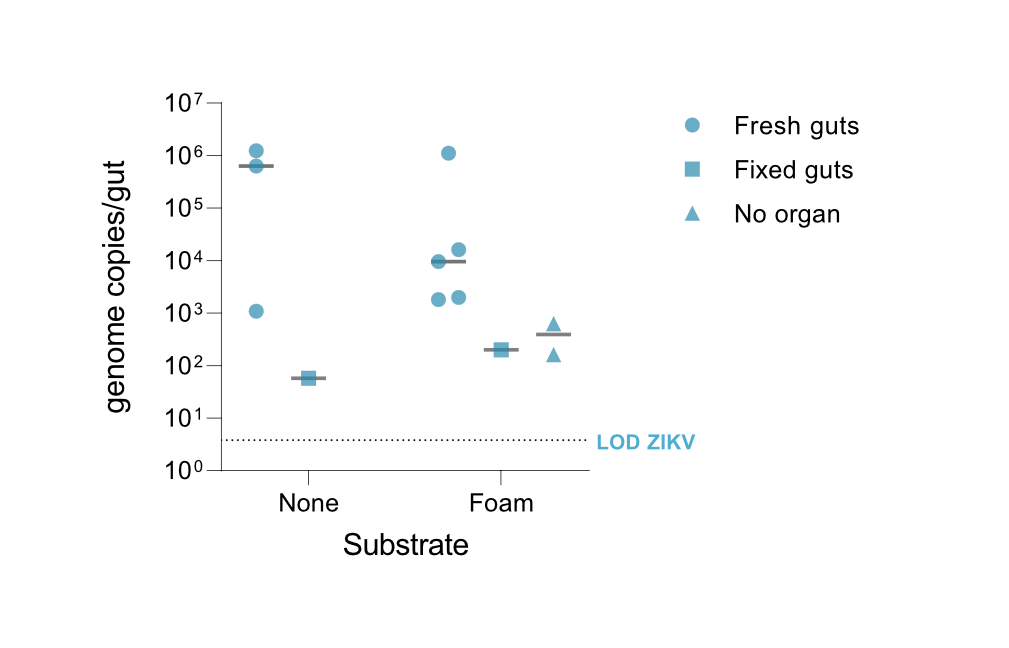


**Figure S5**. **ZIKV RNA loads in the mosquito guts when cultured in medium or when using a foam substrate for support.** RNA levels were quantified at day 7 p.i. by qRT-PCR. The black line represents the median value. Fresh guts: alive guts, negative control: PFA fixed guts, no organ: foam substrate only. LOD: limit of detection of the assay.


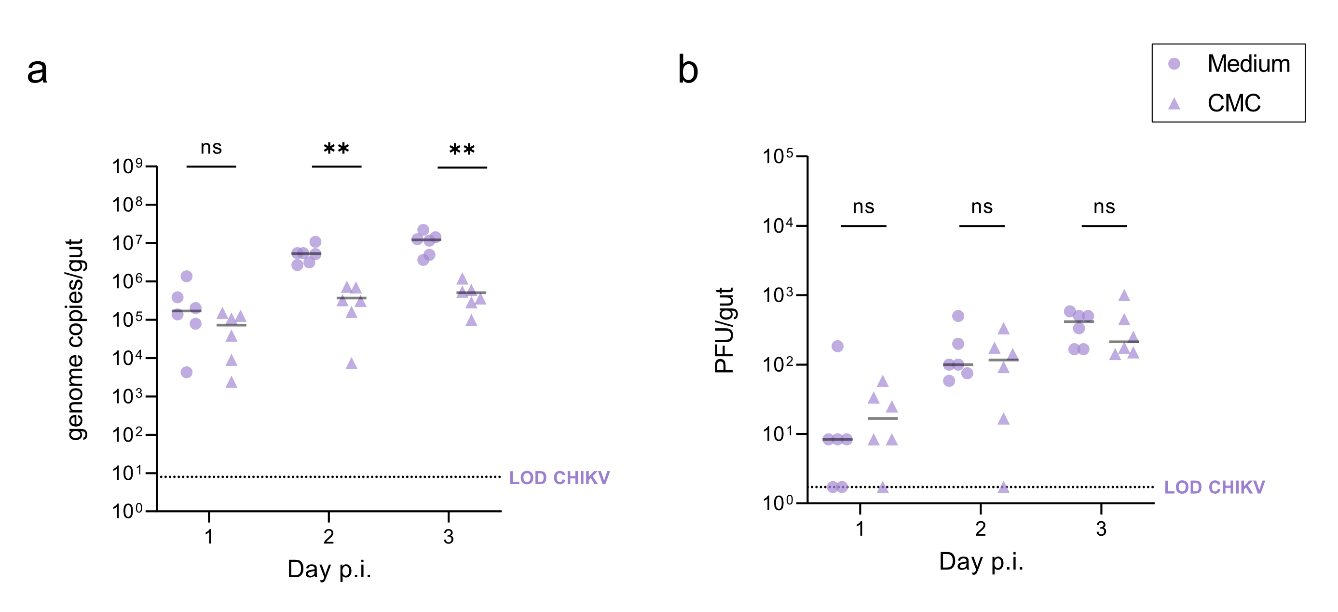


**Figure S6**. **CHIKV replication kinetics in mosquito guts cultured in medium and in carboxymethylcellulose (CMC).** **a**, Viral RNA levels were quantified at day 1, 2 and 3 p.i. by means of qRT-PCR. **b**, Infectious virus loads in the mosquito guts were quantified by means of plaque assay. Each dot represents an individual gut organ. The black line represents the median value. Statistical significance was assessed with a Mann-Whitney test. Significantly different values are indicated by asterisks: **, P<0.005. ns: not significant. LOD: Limit of detection of the corresponding assay.


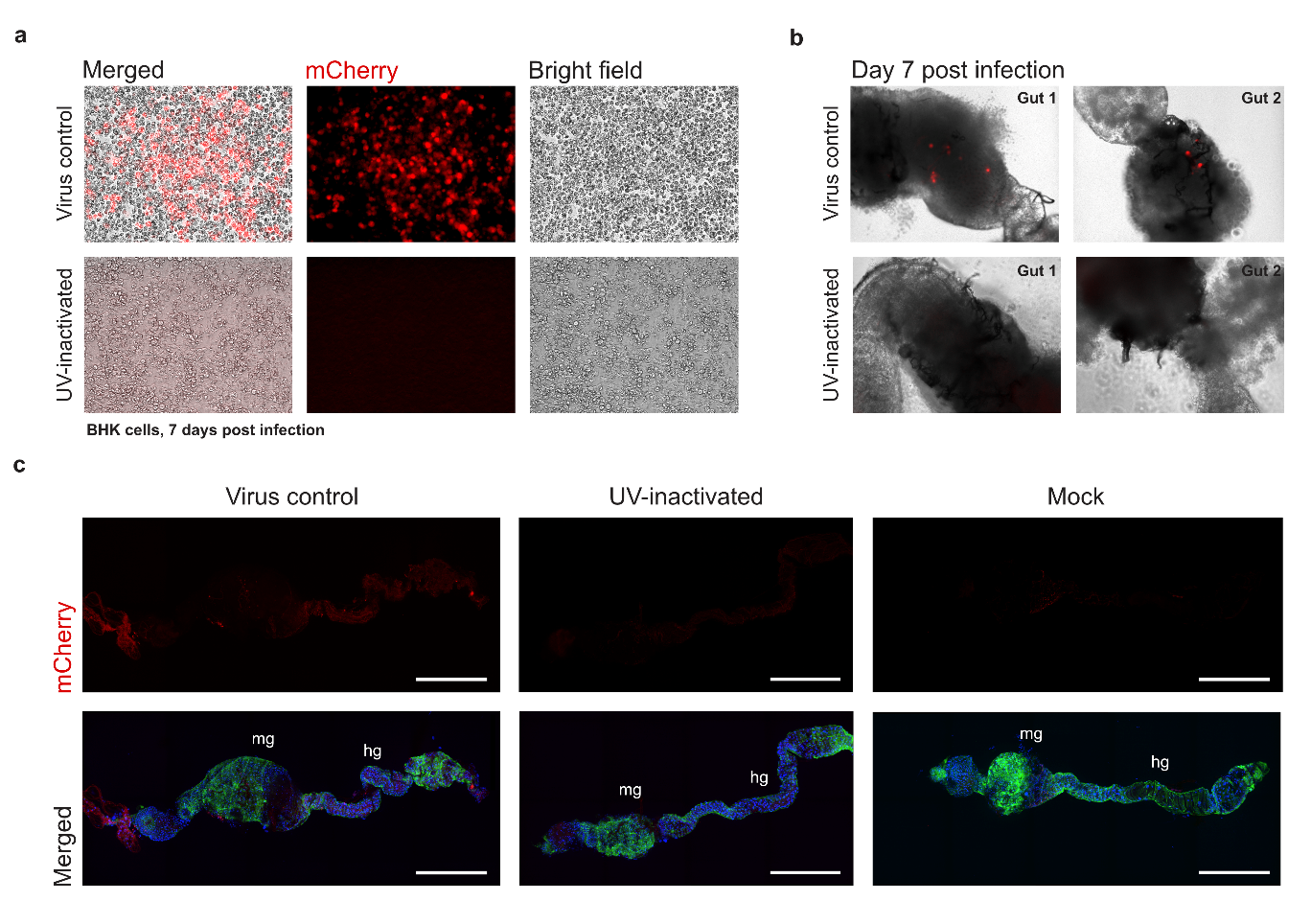


**Figure S7**. **Infection of UV-inactivated DENV-2 expressing mCherry in the *ex vivo* cultured guts.** **a**, Magnification, 20X. Overlay of bright field and red filter imaging, along with each single channel is shown. Panels display the passaging of the DENVmCherry (virus control) and the corresponding UV-inactivated stocks in BHK cells after 7 days of incubation. **b**, Magnification, 20X. Overlay of bright field and red filter imaging. Guts infected with either DENVmCherry (virus control) or the corresponding UV-inactivated virus are displayed. No mCherry signal is detected in guts infected with the UV-inactivated virus at day 7 post infection, compared to the virus control guts. **c**, Magnification, 25X. Confocal microscopy displays the DENV-2 infection in the *ex vivo* guts at day 7 p.i., as seen by the mCherry (red) signal. Overlay of blue, green, and red filter imaging are shown for one gut organ per condition. In blue, DAPI-stained cell nuclei; in green, actin filaments stained with phalloidin; and in red, mCherry signal. No mCherry expression was detected in fixed or mock-infected guts. The scale bar in panels, represented by the white line, corresponds to 400 µM. mg: midgut. hg: hindgut.

**Movie 1.** Representative mosquito hindgut videography used for the hindgut motility analysis. Peristalsis is observed as waves along the hindgut region.
